# Convergent and Divergent Migratory Patterns of Human Neutrophils inside Microfluidic Mazes

**DOI:** 10.1038/s41598-018-20060-6

**Published:** 2018-01-30

**Authors:** Leo Boneschansker, Julianne Jorgensen, Felix Ellett, David M. Briscoe, Daniel Irimia

**Affiliations:** 1 BioMEMS Resource Center, Department of Surgery, Massachusetts General Hospital, Harvard Medical School, Shriners Hospitals for Children, Boston, MA USA; 2 Transplant Research Program and The Division of Nephrology, Department of Medicine, Boston Children’s Hospital, Boston, MA USA; 3 Department of Pediatrics, Harvard Medical School, Boston, MA USA

## Abstract

Neutrophils are key cellular components of the innate immune response and characteristically migrate from the blood towards and throughout tissues. Their migratory process is complex, guided by multiple chemoattractants released from injured tissues and microbes. How neutrophils integrate the various signals in the tissue microenvironment and mount effective responses is not fully understood. Here, we employed microfluidic mazes that replicate features of interstitial spaces and chemoattractant gradients within tissues to analyze the migration patterns of human neutrophils. We find that neutrophils respond to LTB4 and fMLF gradients with highly directional migration patterns and converge towards the source of chemoattractant. We named this directed migration pattern *convergent*. Moreover, neutrophils respond to gradients of C5a and IL-8 with a low-directionality migration pattern and disperse within mazes. We named this alternative migration pattern *divergent*. Inhibitors of MAP kinase and PI-3 kinase signaling pathways do not alter either convergent or divergent migration patterns, but reduce the number of responding neutrophils. Overlapping gradients of chemoattractants conserve the convergent and divergent migration patterns corresponding to each chemoattractant and have additive effects on the number of neutrophils migrating. These results suggest that convergent and divergent neutrophil migration-patterns are the result of simultaneous activation of multiple signaling pathways.

## Introduction

Neutrophils are guided towards their target location within tissues by a broad range of chemoattractants, including bacterial products such as N-Formyl-Met-Leu-Phe (fMLF), complement factors such as component 5a (C5a), tissue derived cytokines such as interleukin-8 (IL-8), and leukocyte-released lipid mediators such as Leukotriene B4 (LTB4)^1–4^. Several of these chemoattractants can be present in a tissue simultaneously. Thus, neutrophils must integrate all signals to migrate effectively. For example, fMLF gradients around bacterial targets are assumed to elicit rapid and directional chemotaxis of neutrophils in the close vicinity of the source^1^. C5a, which is formed when complement component 5 is cleaved by infectious pathogens^5^, is similarly assumed to elicit a rapid, short range chemotaxis response. fMLF and C5a have thus been named end-target chemoattractants. Meanwhile, LTB4 and IL-8 released by multiple cell types during inflammation^4,6,7^ are thought to elicit neutrophil recruitment from larger distances, towards the areas of the tissue under stress. They are often called intermediary chemoattractants. However, it is still not completely understood how neutrophils integrate responses to multiple chemoattractants that they encounter during their migration from blood into sites of inflammation^8–10^. The current paradigm for neutrophil responses to multiple chemoattractants has evolved from observations of their response to opposing gradients and indicate that neutrophils prioritize between different groups of chemoattractants. For example, the responses to fMLF and C5a were found to override responses to LTB4 and IL-8 gradients in opposite directions^9^. The molecular mechanisms underlying this prioritization were proposed to involve P38 MAP kinase signals in the presence of fMLF or C5a, and the activation of the PI-3 kinase/Akt signaling in response to LTB4 or IL-8^9,11,12^. However, this finding is being challenged by reports showing that PI-3 kinase-induced signals are essential for migration to fMLF^13^, and that PI-3 kinase is only partially^14,15^, or not required in fMLF induced migration^9,16^. This suggests that various levels of crosstalk might exist among signaling pathways involved in functional migratory responses to various chemoattractants.

Recent developments in microfluidic technologies have enabled precise quantification of complex migration patterns at the single cell level^17^. Microfluidics can also mimic the biophysical properties of the tissue by recapitulating spatial and temporal events like those that leukocytes encounter *in vivo*^18^. In this manner, microfluidic technologies are instrumental for the study of directional decision-making of migrating leukocytes^19^, measurements of migratory persistence and speed^20^, and characterization of exploratory patterns of migration^21^. Recently, our group has quantified individual migration signatures for various chemoattractants and have shown that well-established molecules including C5a and IL-8 elicit migration both towards and away from the stimulus^20^. However, the precise quantification of migration patterns in the presence of complex gradients of multiple chemoattractants remains technically challenging.

Here, we designed microfluidic mazes that consist of orthogonal channels connected to chemokine reservoirs. The mazes replicate critical features of the interstitial microenvironment within tissues and enable one to quantify neutrophil migration patterns with high precision. Using these new tools, we find that the chemoattractants C5a and IL-8 induce exploratory, *divergent* patterns of migration, and that concentration gradients provide no directional cues. In contrast, we find that LTB4 and fMLF induce highly directional, *convergent* migration patterns, which are precisely aligned with the direction of the gradients. These migratory phenotypes remain unchanged when cells migrate on collagen. Importantly, different concentrations of chemotactic peptides change the fraction of responding (migrating) neutrophils, but do not change the pattern of migration. Furthermore, combinations of chemokines have additive effects and result in a greater influx of cells. Finally, pharmacological inhibitors of either MAP kinase or PI-3 kinase attenuate neutrophil migration towards all chemoattractants but do not selectively promote or inhibit the divergent and convergent migratory signature. In addition, convergent migration induced by fMLF is dependent on intracellular LTB4 synthesis.

## Materials and Methods

### Microfluidic devices

Microfluidic migration mazes were manufactured using standard microfabrication techniques. Briefly, two layers of negative photoresist (SU8, Microchem, Newton, MA), the first 10 µm thin, the second 50 µm thick, were patterned on a silicon wafer by sequentially employing two photolithography masks and photolithography processing cycles. The wafer with patterned photoresist was used as a mold to produce PDMS (Polydimethylsiloxane, Fisher Scientific, Fair Lawn, NJ) parts, which were then bonded irreversibly to standard glass slides (75 × 25 mm, Fisher Scientific). The device consists of an inlet and outlet connected to a main channel for cell-loading with an array of 14 side channel mazes, each ending in reservoirs designed to maintain chemoattractant gradients for more than 15 hours^21^. Cells can migrate from the main channel into the mazes of (350 µm by 550 µm), where they are confined inside 10 × 10 µm channels (cross-section). The distance between nodes was 50 µm. Cells that enter the mazes can be analyzed for their migration in the direction of the chemical gradients (along the y axis) as well as orthogonal exploration (along the x axis). The direct route from the cell loading area towards the chemokine source is a pathway of 650 µm. We analyzed the chemokine gradient using fluorescein 5(6)-isothiocyanate (MW = 1 kDa) and dextran labeled fluorescein (MW = 10 kDa, Thermo Fisher Scientific).

### Neutrophil isolation and migration buffer

Human peripheral blood samples from healthy volunteers, aged 18 years and older, were purchased from Research Blood Components, LLC. Peripheral blood was collected in 10 mL tubes containing a final concentration of 5 mM EDTA (Vacutainer; Becton Dickinson). Nucleated cells were isolated using a HetaSep gradient, followed by the EasySep™ Human Neutrophil Enrichment Kit (STEMCELL Technologies, Vancouver, Canada) per the manufacturer’s protocol. During migration assays, neutrophils were suspended in Hanks Buffered Salt Solution (HBSS, ATCC, Manassas, VA) with 0.2% human serum albumin (HSA, Sigma-Aldrich, St. Louis, MO).

### Device priming and chemoattractant gradient formation

A blunt needle connected to the inlet of the device served as a loading reservoir for chemokines diluted in migration buffer and the cells. The device was primed 15 minutes prior to cell loading with fMLF (Sigma-Aldrich), leukotriene B4 (LTB4, Cayman Chemicals, Ann Arbor, MI), IL-8 and C5a (R&D Systems Inc, Minneapolis, MN) and/or its combinations with fibronectin (120 kDa, Sigma Aldrich, 1 μg/mL, 1:20 dilution) or with collagen (Corning® Collagen I, Corning Incorporated, Corning, NY). After 15 min, the chemokine solution was gently flushed out of the main channel, resulting in the formation of a chemokine gradient inside the maze.

### Neutrophil loading in microfluidic devices with mazes

Next, 10 µl neutrophil suspension in a density of 40 × 10^6^ neutrophils/mL was loaded in the device and allowed to settle at 37 °C for a few minutes before imaging started. In experiments where the P38 MAP kinase pathway was inhibited, cells were incubated with SB203580 (10 μM, Cell Signaling Technology, Danvers, MA) 30–45 min prior to loading. To inhibit the PI3 kinase pathway, we used LY294002 (50 μM, Invitrogen, Frederick, MD). LTB4 intracellular synthesis was inhibited using MK866 (Abcam, Cambridge, MA).

### Device setup for testing the roles of soluble and surface-bound chemoattractants

To test whether chemokines might be bound to the surfaces of our devices after washing, we employed a motility assay that consists of a single, straight, 500 × 70 µm PDMS channel bound to a glass-bottom well plate. The channel was primed with chemoattractant and fibrinogen in media, incubated at 37 °C for 15 mins, washed thoroughly with media, then the entire device covered in media. Fluorescence imaging of FITC-labelled IL-8 before and after the washing was employed to validate the efficiency of washing protocol for removing the chemoattractant from inside the channel, including the corners of the channel.

Neutrophils were loaded as a suspension in a density of 20 × 10^6^ neutrophils/mL. After loading, neutrophils were allowed to settle at 37 °C for a few minutes before imaging started. The trajectories of the neutrophils were plotted with reference to their starting position. The velocity of neutrophils was calculated by automated cell tracking of cell movement (TrackMate, Image J) during time-lapse imaging.

### Imaging and data analysis

Time-lapse microscopy was performed on a fully automated Nikon TiE microscope with bio-chamber heated to 37 °C, 5% CO_2_ using NIS-Elements AR 3.10 software (Nikon Instruments Inc, Melville, NY). Images were acquired every 40 seconds for 3 hours, using a 10X objective. Cells were tracked manually using ImageJ software (NIH). A custom algorithm was then used to map each individual migration trajectory and determine the migratory speed, directionality and persistence of each moving neutrophil. Next, the traffic along each individual node was calculated. From these nodal maps, a “heat map” was generated in MATLAB (MathWorks, Natick, MA), which provides analysis of node passage by the neutrophils in each condition (and thereby area of maze coverage). The percentage of cells migrating was partially manually counted by determining the number of cells that were at T = 0 within 100 µm of the maze entrance. The migratory persistence was calculated by using the following formula: length of the maze/total distance travelled by the cells x the position of the cell when it stopped migrating as a relative number with entrance of maze being 0 and the end of the maze (reservoir) being 1. Nodal maps were visualized with a “heatmap” generated in Python (Python Software Foundation, Wilmington, DE). The bivariate Kernel Density Estimation (KDE) of cell position was plotted with Seaborn KDE plot using a Gaussian kernel.

### Statistical analysis

For most comparisons, statistical significance was determined using the student t test for normally distributed data and the Mann Whitney test for data that did not have normal distribution. Differences were considered statistically significant when p values were less than 0.05.

## Results

### A microfluidic maze replicates chemical and mechanical features of interstitial space

**Figure 1:**
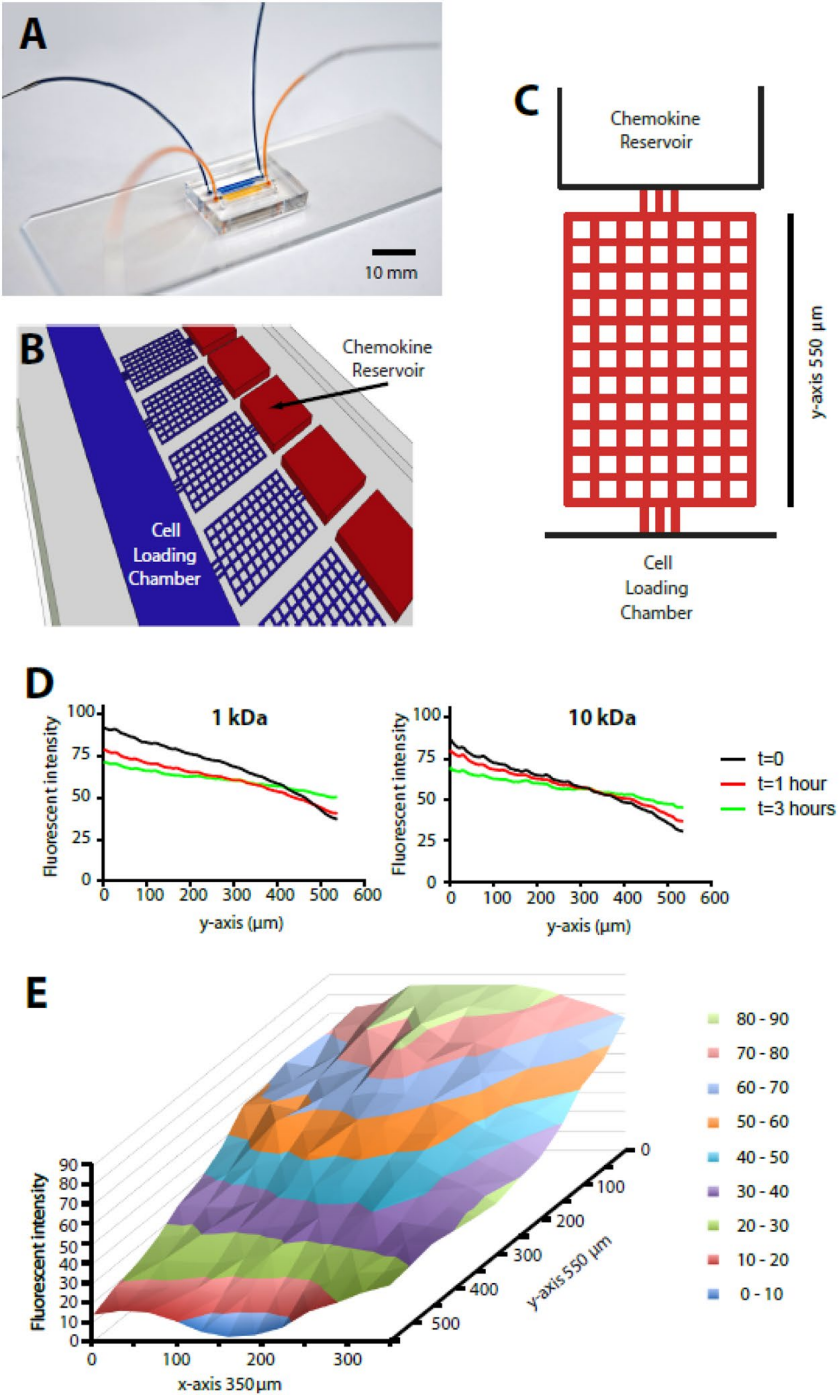
Microfluidics mazes to mimic the complexity of navigating through heterogeneous tissue microenvironments. (**A**) Image of the microfluidic device bound to a standard glass slide. Scale bar represents 10 mm. (**B**) Multiple mazes are connected to a single cell loading chamber, from where cells initiate their migration. The mazes are connected to a reservoir that generates a persistent chemokine gradient. (**C**) Schematic overview of the maze simulating the interstitium. The maze consists of 96 nodes 50 µm apart through which the cells can traffic, allowing cells to take different routes towards the source of chemoattractant. (**D**) The fluorescent intensity of a gradient inside the device persists for >3 hours for low molecular weight chemokines (<1 kDa, left panel) and for ~10 kDa chemokines (right panel). The gradient along the maze is visualized for t = 0, 1 and 3 hours. (**E**) The chemokine concentration is the lowest at the entrance of the maze (blue area with fluorescent intensity 0–10) and increases in a linear manner along the y-axis.

We designed a microfluidic device to study the migratory patterns of human neutrophils through orthogonal mazes, towards reservoirs of chemoattractants (Fig. 1A,B). The mazes mimic the confinement found in interstitial spaces by restricting the migration of neutrophils through channels with 10 × 10 µm cross-section^18^. Cells within the cell-loading chamber migrate towards sources of chemoattractant through mazes that are 550 µm long and 350 µm wide (Fig. 1C). Motile neutrophils are mechanically confined and their average migration speed is similar to that recorded *in vivo*^22^. We probed the evolution over time of the chemoattractant gradients for chemokines with a molecular weight less than 1 kDa (LTB4 and fMLF) and ~10 kDa (C5a and IL-8). We found that each gradient persists for more than 3 hours and is linear along the y-axis (Fig. 1D). On the x-axis, higher chemoattractant concentrations are present in the middle of the maze and lower concentration along the periphery (Fig. 1E). The migration trajectories of individual cells in the maze are composed of persistent migration segments along the straight channels^23^ and discrete directional decisions at the nodes^19^. This decoupling of persistence and directional decisions allows for precise quantification and comparisons of migration patterns in the presence of a chemoattractant. Moreover, the device is ideal to differentiate a directed migration pattern (when cells traverse the maze along the long axis towards the chemokine reservoirs) from a random migration pattern (when cells explore the maze in the transversal direction).

### Convergent migration patterns induced by fMLF and LTB4

**Figure 2:**
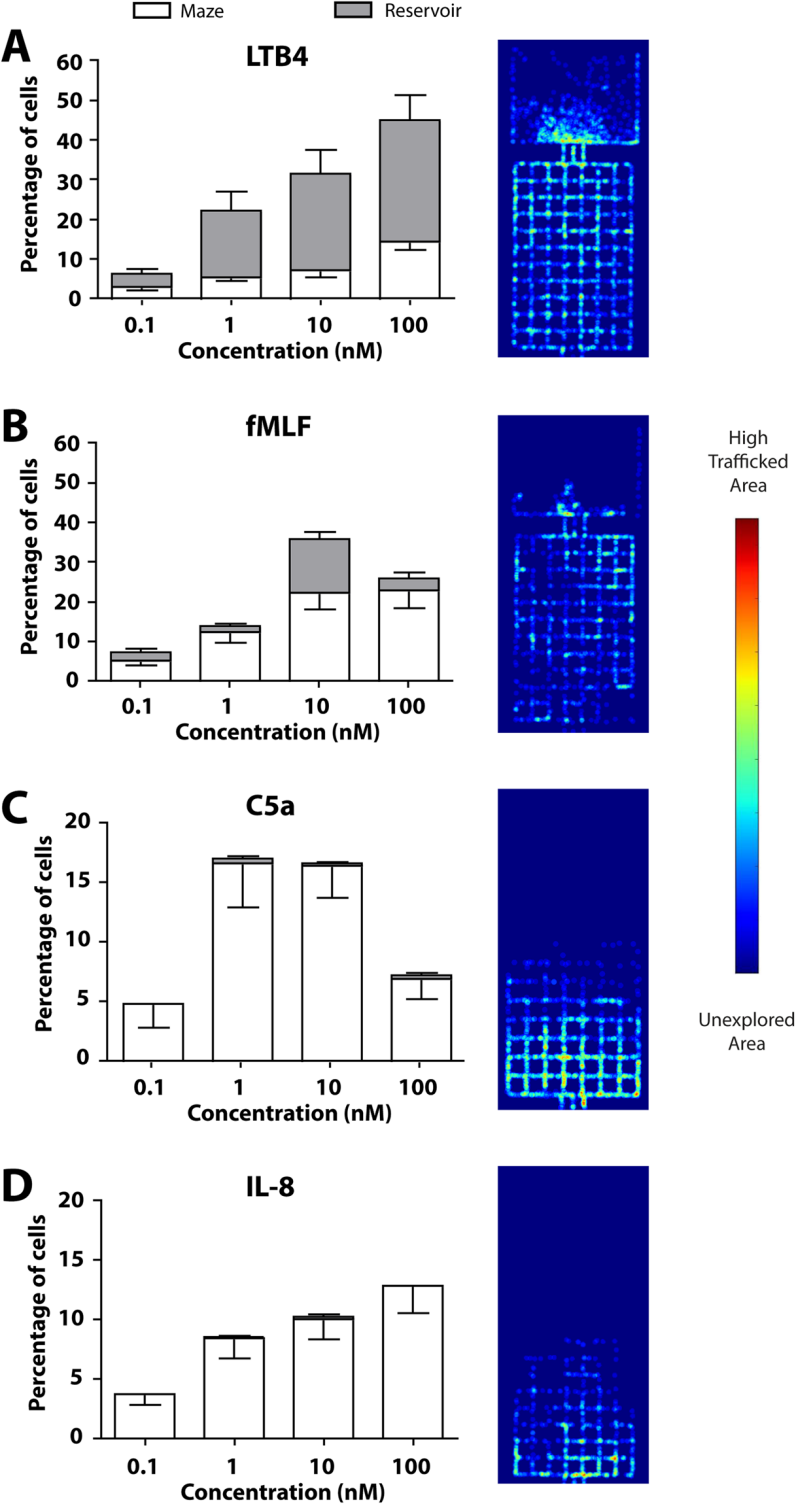
Convergent and divergent migration patterns induced by typical chemoattractants. (**A**) LTB4 elicits a strong directional migratory response at different chemokine concentrations. The bars represent the percentage of cells that migrated within 3 hours. White bars stand for neutrophils ending up in the maze and gray bars show cells migrating all the way through the maze, to the chemokine source. Total analyzed migrating cells is N = 1677. A heatmap representative of 3 hours migration shows most migratory activity around the chemokine source. (**B**) Migratory endpoints induced by fMLF at increasing concentrations. N = 1007 cells analyzed. C5a (**C**) and IL-8 (D) only minimally attract neutrophils to the chemokine source, regardless of concentration used. For C5a N = 414 and IL-8 N = 380 migrating cells were analyzed. Bars represent mean ± SEM. Heatmap documents the result of divergent migration patterns at the entrance of the maze. Graphs represent combined data of N = 3 independent experiments, and 10 nM concentration.

We analyzed human neutrophil responses to LTB4 and fMLF, two chemoattractants which induce a “fast and persistent” migration signature in straight channels^20^. Here, we find that both chemoattractants stimulated the migration of neutrophils into the mazes and their subsequent migration towards the chemoattractant reservoirs. LTB4 elicits a potent chemoattractant response when neutrophils are exposed to gradients ranging from 0.1–100 nM (Movie 1). After 3 hours, 24 ± 22% of the cells loaded into the device completely traverse the maze into the chemokine reservoir, while 7 ± 6% remain in the maze. This overall migration pattern was consistent with most migrating cells (73 ± 20%) moving in a directed manner towards the chemokine source (10 nM, Fig. 2A). The percentage of migrating cells increased from 6 ± 5% to 45 ± 28% in a concentration-dependent response to the chemoattractant concentration increase from 0.1 to 100 nM.

Similarly, fMLF (gradients of 0.1–100 nM) stimulated neutrophils in a manner such that they traverse mazes into the reservoirs (13 ± 7% in the reservoirs vs. 22 ± 16% remaining in the mazes, 10 nM). The fraction of cells that migrate towards the chemokine source was lower than that observed in response to LTB4 (42 ± 19%, p < 0.001) and was concentration-dependent from 0.1 to 100 nM. Notably, we observed that the highest neutrophil response occurred at a concentration of 10 nM. The number of neutrophils migrating decreased by ~10% in response to 100 nM (vs. 10 nM) LTB4 (Fig. 2B).

**Figure 3:**
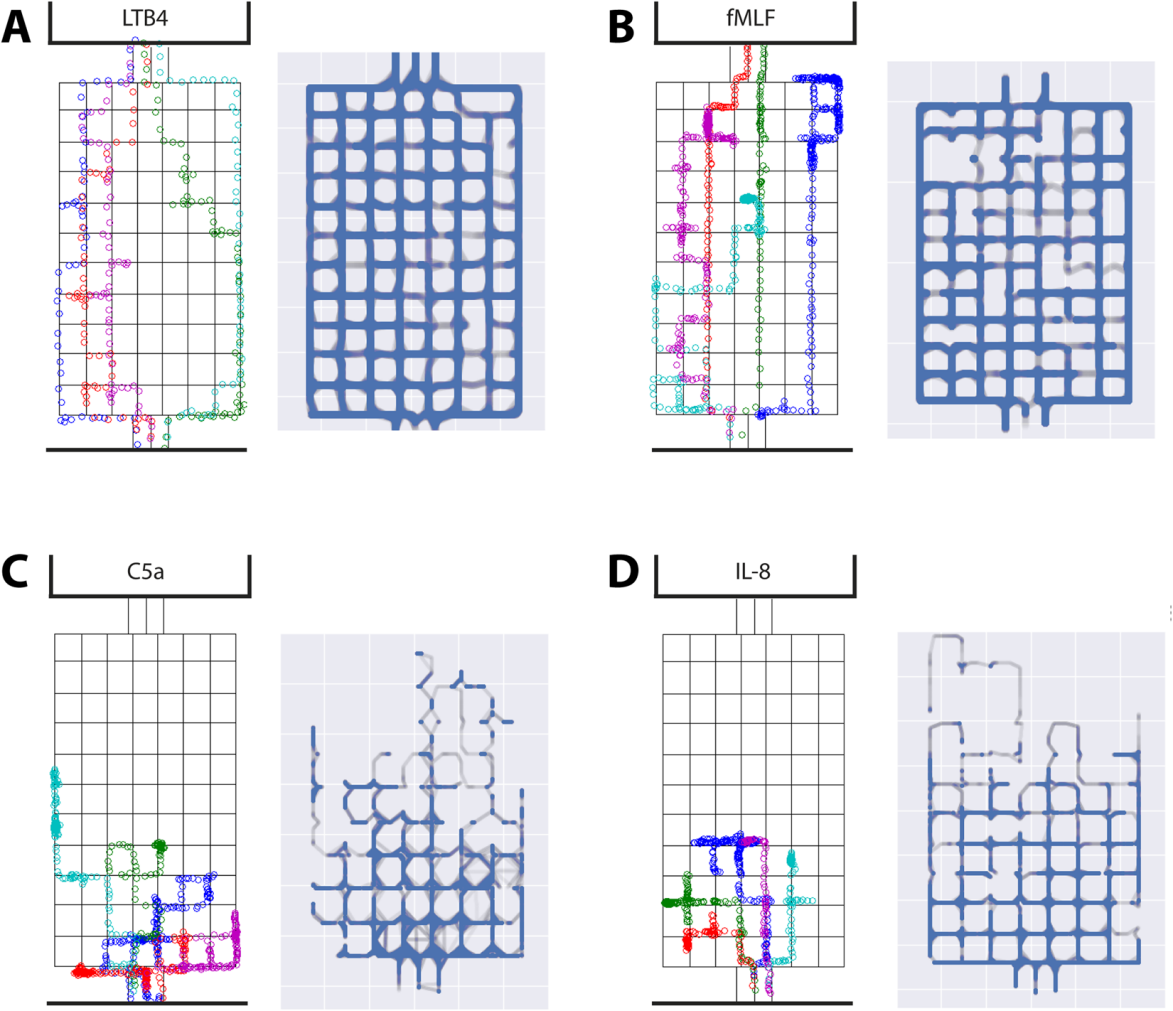
Single cell and average migration trajectories of human neutrophils towards, LTB4, fMLF, C5a, and IL-8 chemoattractants. (**A–D**) Representative trajectories of individual neutrophils encountering gradients of LTB4, fMLF, C5a or IL-8 respectively (10 nM). In the left panels, each color represents a different cell and its position is recorded every 40 seconds. In the right panels, human neutrophil navigation-probability maps representing trajectories that have been traversed by the cells. Travel frequency normalized to the total number of cells in the assay is represented in blue.

**Figure 4:**
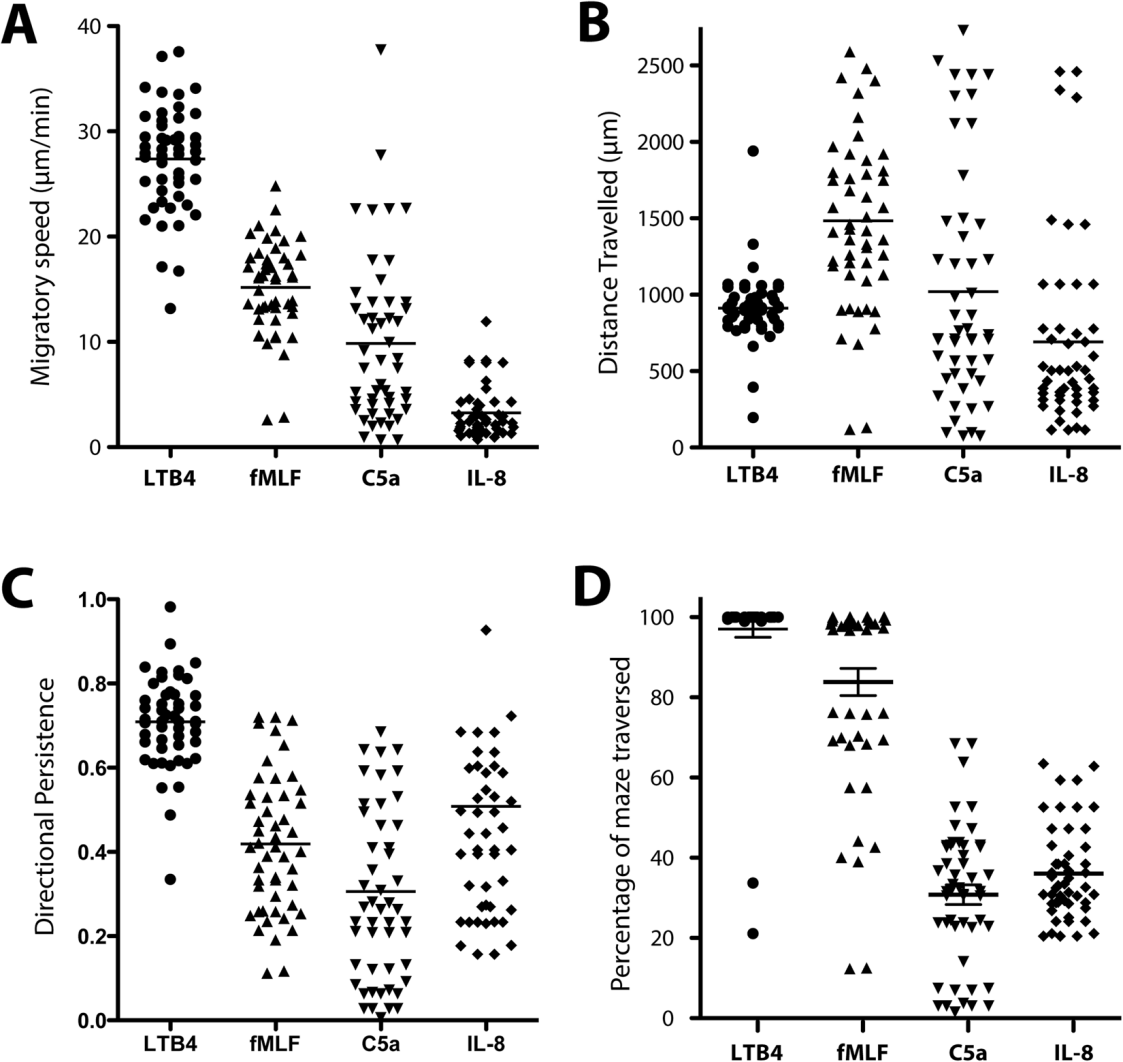
Characterization of migration signatures. (**A**) The average migratory speed of cells while migrating through the maze in response to either LTB4, fMLF, C5a or IL-8 (10 nM). (**B**) The average distance migrated by cells before coming to a final stop in various conditions. (**C**) Directional persistence of migrating cells when encountering a LTB4, fMLF, C5a or an IL-8 gradient (10 nM). (**D**) The percentage of the maze that was traversed by individual cells before it stops migrating. Combined data of N = 50 cells per condition, representative for N = 3 experiments.

Analysis of individual trajectories shows unique patterns for each chemoattractant. Both LTB4 and fMLF stimulate migratory responses with high directionality towards the source (Fig. 3A,B). Analysis of individual cells shows that their average migratory speed was 27.4 ± 5.0 µm/min for LTB4 and 15.2 ± 4.4 µm/min for fMLF (Fig. 4A, p < 0.001). LTB4 also induces transmigration across the maze, within an average time of 37 ± 15 min, covering an average of 912 ± 227 µm during this period, while the shortest route is 650 µm (Fig. 4B). The Directional Persistence of migrating cells is 0.71 ± 0.11 (Fig. 4C). In contrast, neutrophils responding to fMLF do not always migrate along the direct and shortest route (Fig. 3B). The average distance traveled within the maze is 1484 ± 580 µm, the directional persistence is 0.42 ± 0.16, and the time to reach the reservoir is 93 ± 21 min (Fig. 4B,C).

### Divergent migration patterns induced by C5a and IL-8

We analyzed the migratory phenotypes in response to the chemokines C5a and IL-8, which we previously reported to induce bi-directional migration^20^. Although a significant number of neutrophils (17 ± 10% and 10 ± 7%) respond to chemokines in the device, a remarkably smaller number (0.2 ± 0.7% and 0.2 ± 0.8%) show directional migration towards C5a and IL-8, respectively (10 nM, Fig. 2C,D). Overall, we find that these two chemoattractants stimulate exploratory migration (Fig. 3), independent of their concentration. Different concentrations change the number of cells migrating, but not the migration patterns of the cells (Fig. 2C,D). Interestingly, we found that the highest C5a concentrations (100 nM) inhibit migration compared to lower concentrations (Fig. 2C), consistent with a bell shaped curve of cell responses to chemoattractants^24^. Overall, the chemokine C5a (10 nM) stimulated exploratory neutrophil migration within the first 31 ± 17% of the maze, close to the entrance, and IL-8 (10 nM) stimulated exploratory patterns within the first 36 ± 11% of the maze (Fig. 4D, Movie 2). Neutrophils migrated a total distance of 1020 ± 760 and 690 ± 610 µm in response to C5a and IL-8, respectively (Fig. 4B). The average migration speed through mazes was 9.8 ± 7.9 µm/min for C5a and 3.2 ± 2.4 µm/min for IL-8, which is significantly slower than the velocity induced by LTB4 or fMLF, 27.4 ± 5.0 and 15.2 ± 4.4 µm/min, respectively (Fig. 4A, p < 0.001).

### Chemokines do not bind to device surfaces and do not induce neutrophil motility

**Figure 5:**
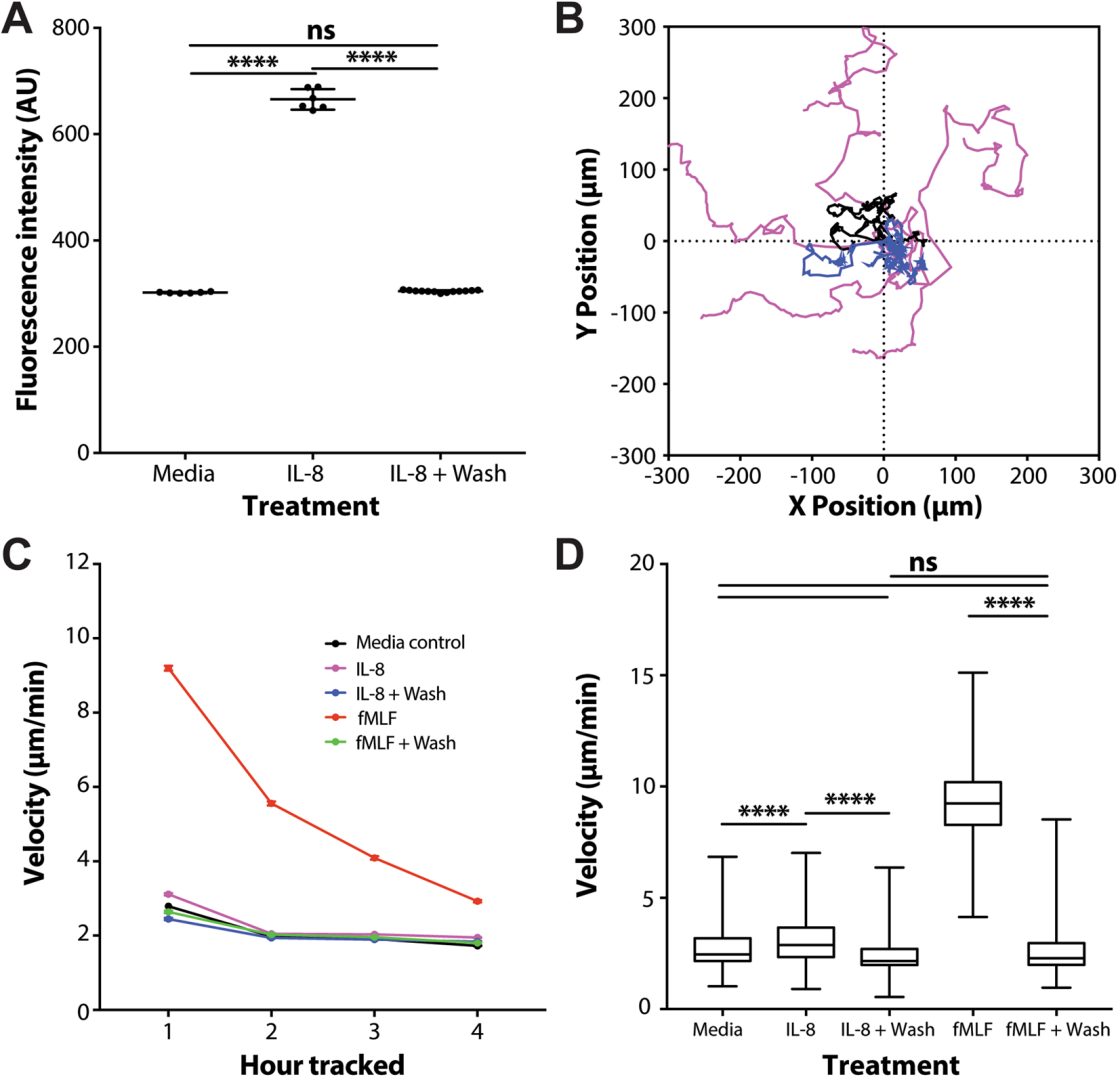
Neutrophils predominantly respond to soluble chemoattractants vs. surface-bound chemokines inside microfluidic devices. (**A**) Graph demonstrates washing efficiently removes FITC-IL-8 from PDMS and glass surfaces. N = 7 FOV for Media and IL-8, N = 14 FOV for IL-8+ Wash. Error bars: mean ± SD. (**B**) Representative tracks from 4 hours of time-lapse imaging of neutrophils in channels containing Media (black tracks), IL-8 (magenta tracks) and IL-8+ Wash (blue tracks). N = 5 tracks per condition. (**C**) Graph shows average neutrophil velocities over 4 hours of time-lapse imaging. Soluble fMLF and IL-8 induce higher neutrophil velocities, which decline over time. Error bars: Mean ± SEM. (**D**) Box plot showing neutrophil velocities in the first hour of tracking. Soluble IL-8 and fMLF induce significantly higher migration velocity than the media control and washed channels. Neutrophils in washed channels did not exhibit significantly higher velocities than the media control (N > 90 cells per condition from N = 3 experiments).

Fluorescence imaging of FITC-labelled IL-8 before and after washing the straight channel devices showed that no fluorescent signal remained following washing compared to the media control. This suggests that FITC-IL-8 was not bound to the channel surfaces in any significant amounts (Fig. 5A). Tracking of neutrophil motility over 4 hours demonstrated that soluble IL-8 was sufficient to induce significant random migration of neutrophils, as indicated by increased migration velocity in the first hour of tracking (Fig. 5D). However, the motility of neutrophils in washed channels was indistinguishable from media control (Fig. 5B–D), suggesting that soluble IL-8 plays a dominant role in the neutrophil motility response. Similar changes in neutrophil motility were measured when comparing the response to soluble fMLF before and after washing the channel. Whereas soluble fMLF induces significant migration of human neutrophils, after the fMLF wash the motility of neutrophils was indistinguishable from the media control (Fig. 5C,D).

### Exposure to collagen gels does not change neutrophil migration patterns in channels

**Figure 6:**
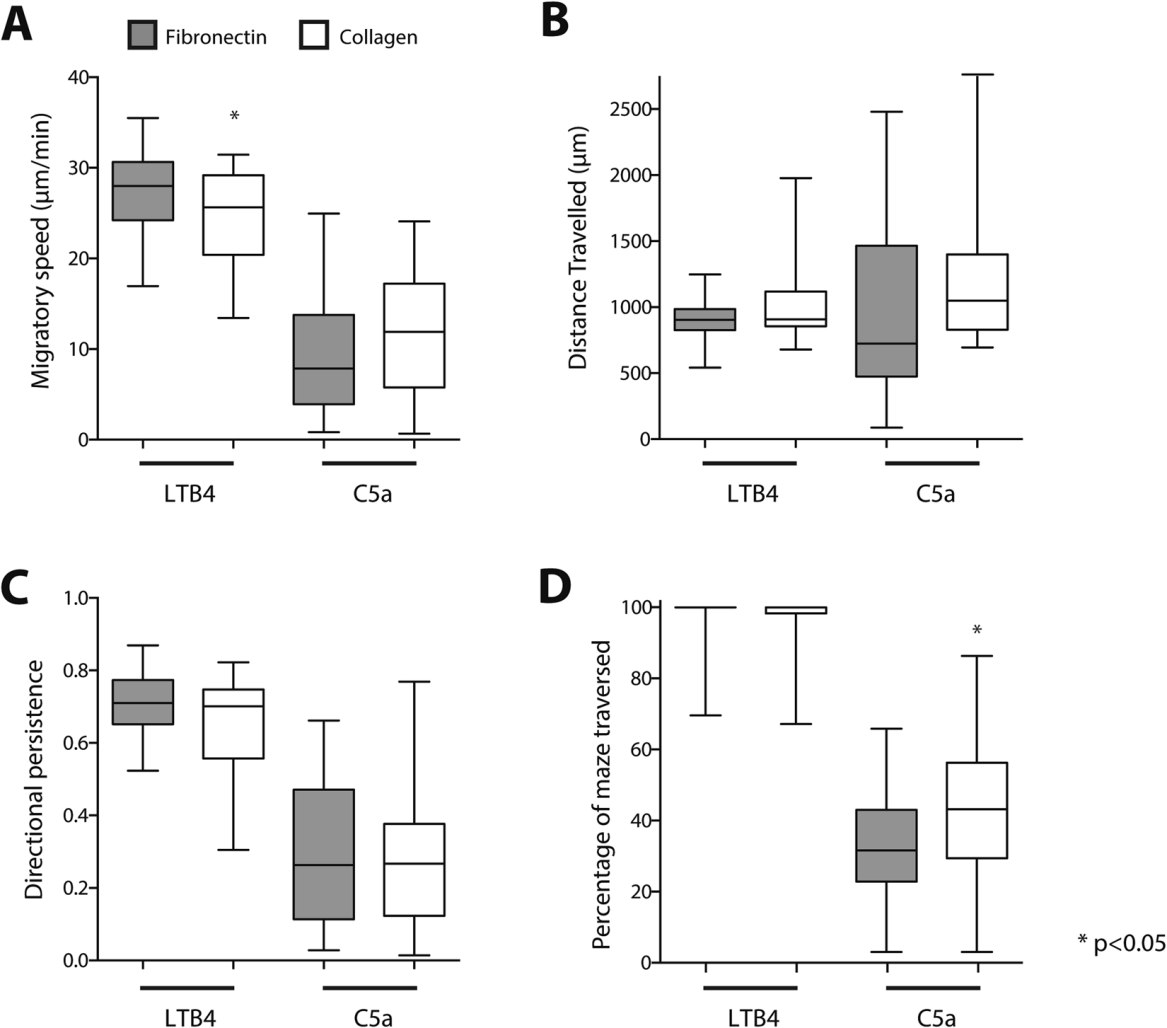
Effect of migratory environment on migration characteristics. The migratory characteristics of neutrophils migrating in the presence of collagen (white bars) where compared with standard migration through fibronectin coated channels (grey bars) while responding to LTB4 (10 nM) or C5a (10 nM). Migratory characteristics that were analyzed were (**A**) migratory speed, (**B**) distance migrated by cells before coming to a final stop, (**C**) directional persistence of migrating cells and (**D**) the percentage of the maze that was traversed by individual cells. The box and whiskers show 5–95 percentile and represent combined data of N = 50 cells per condition, representative for N = 3 experiments.

To determine if migratory patterns change in a true 3D environment, we filled the microchannels with a collagen suspension (rather than coating the channels with fibrinogen). Subsequently, we evaluated the migratory signature, migratory speed, and persistence in this environment. Neutrophil migration speed in response to LTB4 and C5a was similar when collagen was present inside the channel to that observed in fibrinogen coated channels. We measured only a small decrease in the migratory speed in response to LTB4, from 27.4 ± 5.0 to 24.2 ± 5.4 µm/min (Fig. 6A, p < 0.05). The patterns of migration were similar towards LTB4 (persistent migration) and C5a (exploratory migration - Fig. 6C,D). Furthermore, neutrophils that migrated through collagen in response to C5a explored larger areas of maze compared to fibrinogen-coated channels, representing 44 ± 23% and 31 ± 17%, respectively (Fig. 6D, p < 0.05). These migratory phenotypes are comparable to those on bare glass (data not shown).

### Combinations of chemokines show additive migration signatures

**Figure 7:**
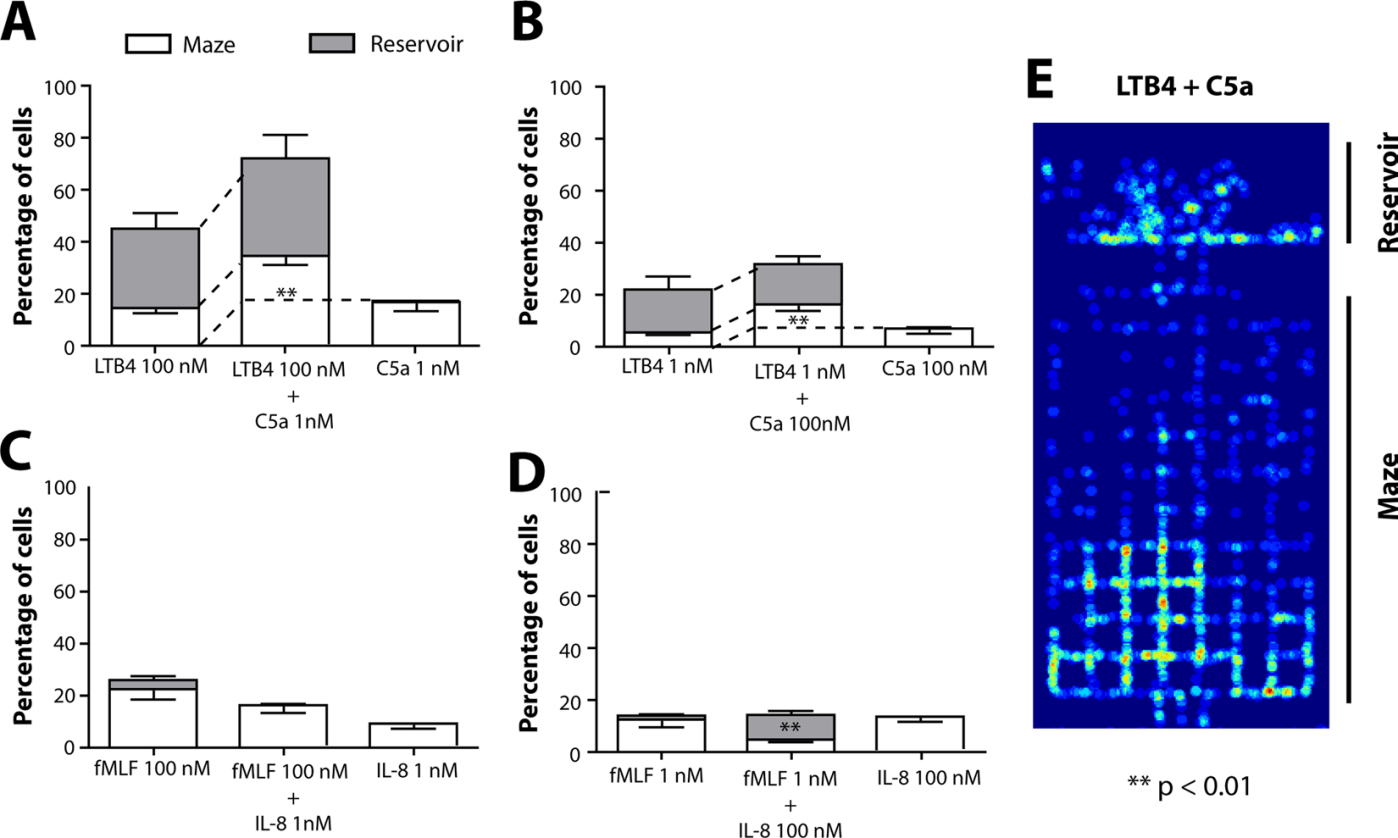
Consequences of chemokine combinations on migration patterns through microfluidic mazes. (**A,B**) Combinations of different LTB4 and C5a concentrations show a migratory pattern that resembles the sum of each individual chemokine. (**C,D**) Combinations of fMLF and IL-8 enhance the convergent migration towards the reservoir, even though the total number of cells migrating is not increased. Bars represent percentage of cells migrating within 3 hours, with white bars representing neutrophils that just enter the maze and grey bars representing neutrophils that migrate through the maze all the way to the chemokine reservoir. Bars represent mean ± SEM and represent combined data of N = 3 independent experiments. Total number of analyzed cells in combined group for A is N = 1072, for B is N = 276, for D is N = 98 and for E is N = 101 migrating cells. (**E**) Heatmap shows increased migratory activity at the entrance of the maze and in the chemokine reservoir (LTB4 and C5a, 10 nM).

We measured the effect of combinations of chemokines on human neutrophil migration through mazes and their ability to reach the reservoir. In response to combined LTB4 (100 nM) and C5a (1 nM) chemokines, there was an increase in the number of neutrophils entering the maze, from 15 ± 8% and 17 ± 12% with each chemokine alone, up to 34 ± 13% in response to combinations (Fig. 7A, Movie 3). Similarly, 31 ± 6% of neutrophils enter the reservoir in response to LTB4 (100 nM) vs. 0 ± 1% in response to C5a (1 nM). This is is comparable to the 32 ± 9% response in the presence of combined LTB4 and C5a (Fig. 7A). We observed similar changes in migratory neutrophil populations in response to combinations of low concentrations of LTB4 (1 nM) with high concentrations of C5a (100 nM, Fig. 7B, p < 0.01). Heat maps confirmed the additive responses. Superimposed migration patterns - inside the maze and reservoir – are consistent with the combined migratory signatures of LTB4 and C5a (Fig. 7E). We did not observe an additive effect during the neutrophil response to combinations of fMLF (100 nM) and IL-8 (1 nM) (Fig. 7C). Mixing lower concentrations of fMLF (1 nM) with higher concentrations of IL-8 (100 nM) enhanced directed migration towards the chemokine reservoir (Fig. 7D, p < 0.01).

### Distinct migratory patterns are not linked to any signaling pathway alone

To determine whether select signaling pathways are responsible for either divergent or convergent migratory behavior we used pharmacological inhibitors to selectively target the P38 MAP kinase (SB203580, 10 µM) or the PI-3 kinase (LY294002, 50 µM) pathways, as previously described^9^. We found that these concentrations of SB203580 (10 µM) and LY294002 (50 µM) used in this assay were nontoxic, and cells migrated in response to agonists.

**Figure 8:**
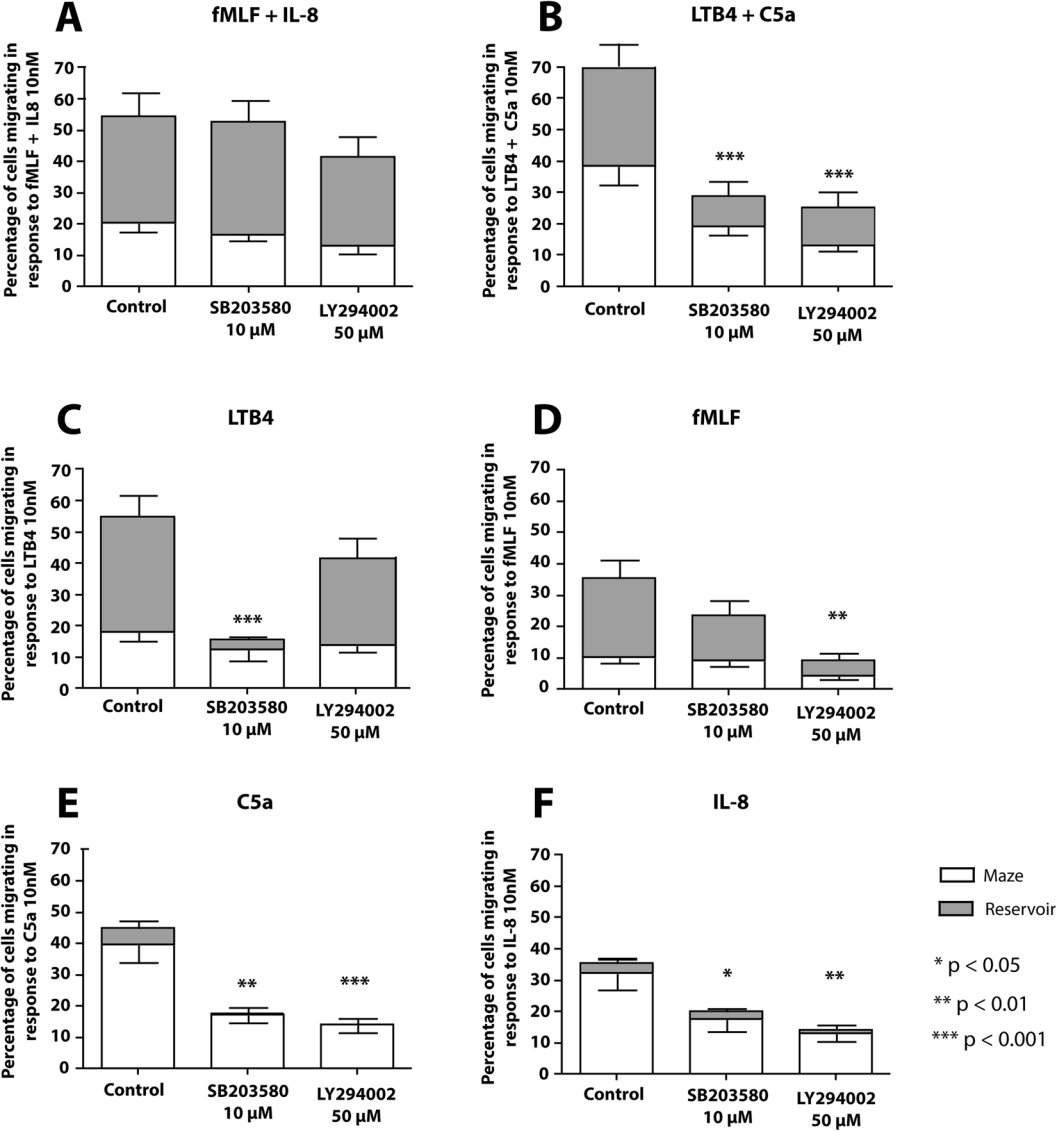
Effect of MAP kinase and PI3 kinase inhibitors on human neutrophil migration towards single and combinations of chemoattractants. Migration patterns were analyzed in the presence or absence of the p38 MAP kinase inhibitor SB203580 or the PI3 kinase inhibitor LY294002 while cells responded to (**A**) fMLF (10 nM) combined with IL-8 (10 nM), (**B**) LTB4 combined with C5a (10 nM), (**C**) LTB4 alone (10 nM), (**D**) fMLF (10 nM), (**E**) C5a (10 nM) and F) IL-8 (10 nM). Bars represent mean ± SEM and are data combined of N = 3 experiments run in duplicate.

Human neutrophil migration towards combined fMLF and IL-8 gradients was not inhibited by neither SB203580 nor LY294002 (54 ± 8% vs. 53 ± 8% and 42 ± 9%, Fig. 8A, P > 0.05). The numbers of neutrophils displaying divergent vs. convergent migratory patterns also did not change (Movie 4, Fig. 8A). Neutrophil migration in response to combinations of LTB4 and C5a significantly decreased, from 70 ± 4% down to 30 ± 4% and 26 ± 6% for SB203580 and LY294002, respectively, p < 0.001 (Movie 5). Moreover, both divergent and convergent migratory patterns were attenuated, regardless of the signaling pathway that was inhibited (Fig. 8B). Finally, we evaluated the effect of inhibitors of P38 MAP kinase and PI-3 kinase on neutrophil migration through mazes in response to single chemoattractant gradients. We found that SB203580 significantly inhibited LTB4 (10 nM)-induced migration, by decreasing the total fraction of migrating cells from 55 ± 6% to 15 ± 4%, and the fraction of neutrophils reaching the chemokine source from 37 ± 7% to 3 ± 1% (Fig. 8C, p < 0.001). LY294002 also reduced migration, but this effect did not reach statistical significance (55 ± 6% vs. 42 ± 5%, Fig. 8C). However, LY294002 reduced the fraction of migrating neutrophils in response to fMLF, from 35 ± 7% down to 9 ± 3% (Fig. 8D, p < 0.01), whereas SB203580 did not significantly decrease the percentage of migrating cells (35 ± 7% vs. 24 ± 5%, Fig. 8D). Inhibition of either the P38 MAP kinase or the PI-3 kinase pathway significantly reduced the fraction of neutrophils entering mazes in response to C5a and IL-8 (Fig. 8E,F, p < 0.05).

### Inhibition of LTB4 synthesis reduces the number of migrating neutrophils

**Figure 9:**
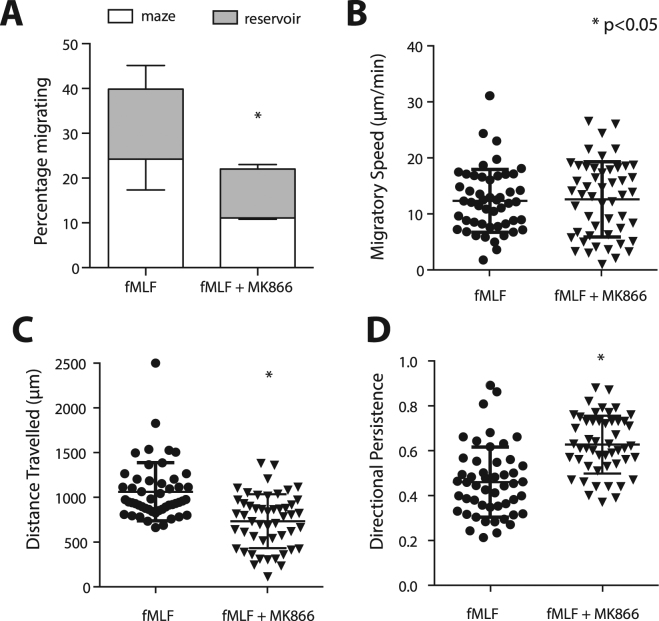
Inhibition of LTB4 signaling reduces migration in cells responding to fMLF. (**A**) Inhibition of LTB4 signaling with MK866 significantly reduced the number of cells responding to an fMLF gradient. Inhibition of LTB4 signaling did not decrease the migratory speed of neutrophils (**B**), but the inhibition significantly reduced the distance travelled before coming to a final stop (**C**) and increased the directional persistence of migrating cells (**D**). Bars represent mean ± SEM and are data combined of N = 3 experiments. The dot plots have the average and standard deviation included and represent combined data of N = 50 cells per condition, representative for N = 3 experiments.

Neutrophils are known to secrete chemokines during inflammatory responses which may alter migration patterns. For example, neutrophils produce LTB4, which plays an important role in swarming^4,7^. Since we observe that LTB4 induces convergent migration, we explored whether LTB4 also potentiates this pattern of migration when other chemokines form the primary gradient. We tested the effects of LTB4 synthesis inhibitor MK866 on neutrophils migrating towards fMLF. Compared to our control experiments, inhibiting LTB4 decreases the percentage of cells migrating from 39 ± 12% to 22 ± 1% (Fig. 9A, p < 0.05). The migratory speed of the neutrophils did not change (Fig. 9B) However, there was a significant reduction in the total distance traveled by neutrophils before coming to a stop, from 1073 ± 340 to 741 ± 300 µm (Fig. 9C). The directional persistence of migrating neutrophils towards fMLF increases from 0.46 ± 0.16 to 0.63 ± 0.13 when LTB4 synthesis was inhibited (Fig. 9D).

## Discussion

We employed microfluidic devices with 2D orthogonal mazes to quantify the migration patterns of human neutrophils towards various chemoattractants. The confinement of moving cells in small channels inside the devices allows the decoupling of the measurements of speed and persistence (along the straight channels), from directional decisions (at the nodes). This is unlike the migration on flat surfaces (e.g. micropipette, under-agarose, Zigmond chamber, Dunn assays), when neutrophils change continuously both their speed and their direction of migration. Instead, the migration through mazes takes place at constant speed^23^ and the direction changes are discrete i.e. neutrophils migrate through one of four orthogonal channels at most nodes^25^. The particularities of migration through mazes helps decrease the noise of measurements and increase the precision of measurements^19,25^ compared to both traditional and other microfluidic chemotaxis devices^26^. Thus, the maze assay enables unprecedented levels of precision for the measurements of speed, directionality, and persistence of the moving neutrophils and helps discriminate between migration signatures that could have not been distinguished by other assays.

Our studies provide insights into human neutrophil migration by showing that two patterns exist, divergent and convergent. These migration patterns may be relevant to the variety of roles that neutrophils play during inflammatory conditions. Rapid neutrophil convergence to sites of bacterial proliferation and their accumulation in swarms is consistent with the ability of neutrophils to accurately follow gradients of fMLF and LTB4^4,7,27^. The spreading of neutrophils through tissues, which favors the clearance of bacterial debris and enhances the surveillance of tissues under stress is consistent with the measured neutrophil divergent motility signature in the presence of C5a and IL-8. The fact that neutrophils retain both migration signatures in response to combined stimuli may be explained by the presence of subgroups of neutrophils. This possibility is supported by recent results documenting the presence of distinct neutrophil subpopulations during inflammation^28^ and within cancer tissues^29^. An alternative explanation is that neutrophils exposed to combined stimuli make one stochastic choice between the stimuli and subsequently maintain one migration signature. This possibility is supported by observations of stochastic responses of neutrophils during exposure to repeated stimuli, when the time to respond and the subpopulation of neutrophils responding changes from one stimulus to the next^30^.

Several studies have focused on the mechanisms of how neutrophils prioritize competing cues^8,9^ and building better tools to study this process^31^. Based on experiments where the migration was evaluated under conditions of opposing gradients, it was proposed that receptor desensitization plays an important role, or that certain chemokines stimulate different signaling pathways^9,32^. Complementing these early studies, we find that chemoattractant gradients in the same direction do not compete with one another and their effects are additive. The conditions we evaluate in this report may be relevant to *in vivo* situations, where neutrophils encounter overlapping gradients of chemokines that originate in the same local area. Our findings also suggest that the two previously proposed, mutually-exclusive signaling pathways may not adequately explain convergent and divergent migration patterns. We could not confirm that C5a elicits discrete signals through the P38 MAP kinase pathway to dominate over the PI-3 kinase pathway activated by LTB4, as suggested in previous studies using opposing gradients^9^. Instead, our data suggest that both the P38 MAP kinase and the PI-3 kinase pathway function in chemoattractant responses. Inhibiting either signaling path alone decreases the size of migratory populations, without altering the pattern of migration. The results of the under-agarose assay used in previous studies are dominated by the number of neutrophils moving, and thus the measured effect may be limited to the size of migratory populations. The under-agarose assay is less sensitive to the patterns of migration, which are the unambiguously decoupled from other changes in our maze assay.

An interesting finding is that in the setting of neutrophil stimulation with two chemotactic peptides (fMLF and IL-8), inhibition of either P38 MAP kinase or PI-3 kinase did not significantly reduce the number of migrating cells. However, when neutrophils encounter fMLF or IL-8 alone, inhibition of either signaling pathway results in significantly lower numbers of migratory cells. It is possible that when two different chemotactic molecules stimulate a cell simultaneously, there is enhanced threshold activity and/or crosstalk between the p38 MAP kinase and PI-3 kinase pathways to overcome the inhibition^33^. These findings again suggest that the two dominant migratory patterns are mechanistically complex and not just the result of discrete signaling through one pathway. The molecular mechanisms responsible for the two distinct migration patterns remain to be elucidated in future studies.

Both convergence and divergence patterns of migration appear to be relatively independent of the concentration of the chemoattractant. Exploratory patterns of neutrophil migration in response to IL-8 have been previously observed *in vivo*^34^. Although these migration patterns were proposed to be due to the degradation of IL-8 by matrix metalloproteinases, our study suggests that the exploratory migration patterns in response to IL-8 are intrinsic to the neutrophils and independent of the IL-8 concentration. Moreover, the convergent and divergent migration patterns observed inside the mazes hold true in a 3D environment, when the maze channels are filled with collagen. Our results suggest that the migration pattern and/or signatures are cell and chemokine intrinsic and they are not related to the physical microenvironment surrounding the cells. Finally, we examined the effect of paracrine signaling on migration phenotypes. We found that when LTB4 synthesis is inhibited during neutrophil migration towards gradients of fMLF; the number of migrating cells decrease, the migration paths are shorter, and migratory persistence is increased. These results are consistent with several reports^4,7,27,35^ and suggest that paracrine LTB4 signaling plays an important role in recruiting cells to an inflammatory focus, potentiating the local inflammatory reaction.

Formation of haptotactic chemokine gradients is important for neutrophil migration in response to some chemokines but not others^36^. Although microfluidic devices have been developed specifically to dissect the differential contributions of superimposed soluble and haptotactic gradients to cell migration^37^, this was not possible using our current maze device design. Surface-bound IL-8 has been shown to induce random migration by human neutrophils^38,39^. A simpler device was therefore employed to compare the motility induced by soluble and bound gradients. These experiments helped verify that soluble gradients in our microfluidic devices dominate the neutrophil responses.

In conclusion, we developed a new set of tools that enable single cell resolution, quantitative measurements of neutrophil migration patterns in response to chemoattractants. We describe two distinct migration patterns, which we name divergent and convergent. Furthermore, we find that they are differentially induced by C5a or IL-8 and fMLF or LTB4, respectively. The new microfluidic tools will enable further studies on the requirements for efficient responses to infectious pathogens and/or could lead to new avenues of investigation on the restoration of tissue homeostasis after inflammation.

## Electronic supplementary material

Human neutrophil navigation through maze towards LTB4 10nM

Human neutrophil navigation through maze towards C5a 10nM

Human neutrophil navigation through maze towards LTB4 100nM and C5a 1nM

Human neutrophil navigation through maze towards fMLF 10nM & IL8 10nM in the presence of LY294002 inhibitor

Human neutrophil navigation through maze towards LTB4 10nM & C5a 10nM in the presence of LY294002 inhibitor
